# Alterations of protein composition along the rostro-caudal axis after spinal cord injury: proteomic, *in vitro* and *in vivo* analyses

**DOI:** 10.3389/fncel.2014.00105

**Published:** 2014-04-17

**Authors:** Dasa Cizkova, Françoise Le Marrec-Croq, Julien Franck, Lucia Slovinska, Ivana Grulova, Stéphanie Devaux, Christophe Lefebvre, Isabelle Fournier, Michel Salzet

**Affiliations:** ^1^Laboratoire de Spectrométrie de Masse Biologique Fondamentale et Appliquée, EA 4550, FRE CNRS 3637, Université Lille 1Villeneuve d'Ascq, France; ^2^Laboratory of Cell and Tissue Culture, Institute of Neurobiology, Center of Excellence for Brain Research, Slovak Academy of SciencesKošice, Slovakia

**Keywords:** microglia, proteomic analysis, inflammation, spinal cord injuries, dorsal root ganglion, chemokines, neurotrophic, secreted protein

## Abstract

Based on proteomic analyses we investigated the differences of released molecules in the conditioned media (CM) from the spinal cord central lesion and adjacent rostral and caudal segments at 3, 7, and 10 days after spinal cord injury (SCI), in order to specify the molecular environment within greater extent of tissue damage. Proteins found in CM were analyzed by shot-gun MS using nanoLC coupled to an orbitrap. The results showed some specific proteins at each site of the lesion at 3days. Among the proteins from rostral and lesion segments, some are related to chemokines, cytokines or to neurogenesis factors. In contrast, proteins from caudal segments are more related to necrosis factors. The CM from each spinal segment were used *in vitro*, on microglial BV2 cell lines and DRGs explants, showing a lesion site-dependent impact on microglia activation and DRGs neurite outgrowth. In addition, while naive BV2 cells exhibited insignificant staining for CX3CR1 receptor, the level of CX3CR1 was strongly enhanced in some BV2 cells after their stimulation by CM collected from SCI. The molecular data might correlate with different polarization of activated microglia and macrophages along the rostro-caudal axis following acute injury. This was partially confirmed *in vivo* with CX3CR1 receptor, revealing higher expression in the rostral segment, with potential neuroprotective action. In addition, the neurotrophic factors released from rostral and lesion segments enhanced outgrowth of DRGs explants. Taken together these data suggest that regionalization in terms of inflammatory and neurotrophic responses may occur between rostral and caudal segments in acute SCI.

## Introduction

Spinal cord injury (SCI) represents one of the most devastating forms of trauma, often leading to permanent spastic paralysis in humans (Beattie et al., 2002). After initial primary injury caused by direct mechanical insult, the spinal cord tissue progressively undergoes pathological changes that are associated with secondary damage affecting intact, neighboring tissue (Tator, 1995; Schwab and Bartholdi, 1996). One of the key events of secondary processes is related to the development of acute inflammation characterized by fluid accumulation (edema) and the recruitment of immune cells (neutrophils, T-cells, macrophages, and monocytes) (Schwab and Bartholdi, 1996; Schwartz et al., 1999). In fact, spinal cord microglial cells normally function as a kind of reactive immune cells that begin to respond to signals after pathological stimuli (injury, infection, or tumors) (Ransohoff et al., 2007) and are activated at the lesion epicenter (Kreutzberg, 1996).

Although, it has been suggested that microglia/macrophages can be polarized into M1-neurotoxic or M2-neuroprotective states, and produce a variety of cytokines, chemokines and neurotrophic factors, the mechanisms regulating microglial polarity remain unclear (Aguzzi et al., 2013). In this context recent studies indicate that macrophages can alter their phenotypes and functions according to changes in the spinal cord microenvironment during sub-acute and chronic phase. Thus, SCI triggers excessive inflammatory response mediated by the invasion of predominantly M2 macrophages into and around the central lesion at sub-acute phase, but not at chronic phase that is involved in the formation of glial scar (Nishimura et al., 2013). These findings correlate with accumulated data pointing to a chronological time line expression of different degeneration and regeneration associated genes that are involved in the pathogenesis and endogenous repair or plasticity during days to months following SCI (Gerin et al., 2011). However, not only microglia/macrophages but also astrocytes, meningeal cells and fibroblasts together with the increased production of inhibitory chondroitin sulfate proteoglycans (CSPGs) are involved in the spinal cord pathogenesis (Fitch and Silver, 1997).

Moreover, it seems that complex changes in gene and protein expression as well as in cellular interactions are taking place not only at the central lesion, but also in adjacent segments (above and below central lesion). However, the exact mechanisms of the proteins involved during secondary damage, inflammation, recruitment, and polarization of microglia, activation of astrocytes and glial scaring, remyelination, or axonal growth and plasticity, remain to be further explored. Thus, to better understand the secondary damage processes and plasticity, we used a reliable and reproducible balloon-compression technique to produce SCI (Vanicky et al., 2001). Sham operated vs. SCI rat spinal cord tissues were analyzed at 3 days post-lesion; when the polarization of microglia into M2 phenotype seems to transiently (3–7 days) dominate (Kigerl et al., 2009). The collection of tissues from epicenter and both adjacent segments above (rostral) and below (caudal) the lesion allowed to study released molecules.

We, have taken advantages of proteomic technology to screen and identify peptides in each spinal cord segment-derived conditioned medium (CM), obtained *in vitro*, to better understand protein composition changes along the rostro-caudal axis after SCI with time (3, 7, 10 days). Afterwards, we have used these CM for i*n vitro* tests investigating the BV2 cells activation by chemotaxis assay, western blot, and M1/M2 polarization through CX3CR1 and CD206 expression, based on immunocytochemistry *in vitro* and *in vivo*. Chemotaxis assays showed that BV2 cells were highly responsive to the CM derived from rostral and lesion segments, compared to CM from caudal site. Efficacy of neurotrophic factors released from rostral and lesion segments was confirmed on enhanced outgrowth of DRGs explants.

In summary we demonstrate that at 3 days after SCI, a clear regionalization occurs between the rostral and caudal axes, with expression of neurotrophic and immune modulatory factors in the rostral region, in contrast to inflammatory and apoptotic molecules in the caudal region.

## Experimental procedures

### Chemicals

All chemicals were of the highest purity obtainable. Water, formic acid (FA), trifluoroacetic acid (TFA), acetonitrile (ACN), and methanol (MeOH) were purchased from Biosolve B.V. (Valkenswaard, the Netherlands). Sequencing grade, modified porcine trypsin was purchased from Promega (Charbonnieres, France).

### Animals

The study was performed with approval and in accordance to the guidelines of the Institutional Animal Care and Use Committee of the Slovak Academy of Sciences and with the European Communities Council Directive (2010/63/EU) regarding the use of animals in Research, Slovak Law for Animal Protection No. 377/2012 and 436/2012.

### Spinal cord trauma

The SCI was induced using the modified balloon compression technique in adult male Wistar rats (*n* = 16), according to our previous study (Vanicky et al., 2001). Manual bladder expression was required for 3–10 days after the injury. In the sham group (control, *n* = 16) a 2-French Fogarty catheter was inserted at the same level of spinal cord, but the balloon was not inflated and no lesion was made. SCI animals (*n* = 16) were divided in groups processed for immunohistochemistry (*n* = 4) and for CM production (*n* = 12) with corresponding sham-controls.

### Tissue processing and immunohistochemistry

Rats following SCI (*n* = 4) and sham surgery (*n* = 4) at day 3 were deeply anesthetized and perfused transcardially with saline, followed by 4% paraformaldehyde (PFA) in 0.1 M phosphate-buffered saline. Spinal cords were removed, post-fixed in 4% PFA, embedded in gelatin–egg albumin protein matrix (10% ovalbumin, 0.75% gelatin, glutaraldehyde) and soaked overnight in 30% sucrose. Each spinal cord was dissected into 1.0 cm blocks (lesion site, rostral, and caudal segments to the lesion) and 30-μm thick transverse cryostat (Leica Instruments, Heidelberg, Germany) sections were cut serially (100 μm interval) (*n* = 60 each segment) and standard immunohistochemistry (IHC) technique was performed. Tissue sections were incubated in the following primary antibodies: anti-Iba1 (a marker for microglia/macrophages, rabbit IgG, 1:1000; Wako Pure Chemical Industries, Osaka, Japan), anti-ED1 (a marker for monocytes, mouse IgG,1:500; Chemicon, Millipore, Billerica, MA, USA), anti-CX3CR1 receptor and anti-CD206 (a marker for M2 phenotype, rabbit IgG,1:100; Santa Cruz Biotechnology, Santa Cruz, CA, USA) that after wash in PBS was followed by secondary fluorescent antibodies: goat anti-rabbit IgG conjugated with Texas Red (Alexa Flour 594), goat anti-mouse IgG or goat anti-rabbit IgG conjugated with Oregon Green dye (Alexa Flour 488). Fluorescence conjugated secondary antibodies were purchased from Molecular Probes, Oregon, USA. Omission of the primary antibody served as the negative control. For nuclear staining, we used 4-6-diaminidino-2-phenylindol (DAPI) (1:200). Finally, sections were washed in 0.1M PBS, mounted, and cover slipped with Vectashield mounting medium (Vector Laboratories, Inc.) and observed under a fluorescence microscope (Nikon Eclipse Ti, Japan) and confocal laser scanning microscope (Leica TCS SP5 AOBS, Leica Microsystems, Mannheim, Germany).

### IHC quantification

Quantificative analyses of immunofluorescence staining for Iba1, CX3CR1, and CD206 were performed on 8 sections/per animal (rostrally/caudally, *n* = 4 each; captured fluorescence digital images) at 40× magnification and were analyzed by Image J software according to the previous protocol (Jones et al., 2002). For each SCI and sham group we analyzed totally 32 transverse sections (Iba1+/CX3CR1+/CD206+ *n* = 4). In the monochrome 8-bit images we have determined the mean gray level number of black and white pixels within five identical sampling fields (250 × 250 μm) in following regions: gray matter (summary dorsal + ventral areas), white matter divided into Dorsal, Lateral and Ventral White Matter, and digitally subtracting a background image of a control section from each image. The threshold values were maintained at a constant level for all analyses.

### Collection of CM from control and lesioned spinal cord segments

Experimental SCI rats (*n* = 4) at 3, 7, and 10 days and sham-operated-control rats (*n* = 4) were sacrificed by isoflurane anesthesia followed by decapitation. The spinal cord was pressure expressed by injecting sterile saline (10 ml) throughout the vertebrate canal, along the caudo-rostral axis. Each spinal cord was macroscopically observed and the central lesion distinguished at the Th8-Th9 level. Samples (approximately 1.0 cm each) taken from the central lesion (Th7-Th11) rostral (Th2-Th6) and caudal (Th12-L3) segments to the site of injury were additionally chopped into 0.3 cm thick sections/3 per segment and deposited into a 12-well culture plate containing 1 ml DMEM without fetal calf serum (FCS). After 24 h incubation in a humidified atmosphere with 5% CO_2_ at 37°C, 1 ml of SCI-derived conditioned media CM (CM-SCI) were collected (rostral, lesion, caudal segments) and centrifuged 30 min at 15,000 rpm at 4°C. The same procedure was performed for obtaining CM from sham spinal cord tissue. From the 1 ml stock 50 μL samples were then taken and subjected to trypsin digestion (24 h, 37°C) followed by desalting using C18 ziptips (Millipore). The solution was dried under vacuum and resuspended in water /5% acetonitrile /0.1% formic acid before injecting into nanoLC. Unused samples were stored at −80°C.

### Chemotaxis assays

The effects of CM obtained from control (CM CTR) or injured spinal cord (CM SCI) along the rostral (CM RSCI), lesion (CM LSCI) or caudal segments (CM CSCI) on microglial recruitment were determined using the Boyden chambers (Cell Biolabs, CytoSelect™ 96-Well Cell Migration Assay, 5 μm) (Smith et al., 2008). The BV2 cells (Species: mouse, C57BL/6; Tissue: brain, microglial cells) were purchased from the IRCCS Azienda Ospedaliera, Universita San Martino (Italy) (Bocchini et al., 1992) and initially cultured in Roswell Park Memorial Institute (RPMI) 1640 medium supplemented with 10% FCS and 1% penicillin/streptomycin (P/S), and split twice a week to obtain a sufficient number of BV2 cells. Before experiment, cells were plated in Dulbecco's Modified Eagle's Medium (DMEM) with P/S (all reagents from Invitrogen). Replacement of RPMI with DMEM did not decrease the viability of BV2 cells nor changed their morphological pattern (data not shown). In the first experiment, BV2 cells at a concentration of 50,000 per insert were placed into the upper chamber while the CM from control and from different segments (rostral, lesion and caudal) of the injured spinal cord were filled into the lower one (1:3, CM: DMEM) and then cultured for 3 h. Prior to application, the CM from each spinal segment was centrifuged 10 min at 1500 rpm and sterilized with 0.22 μm filters. The protein concentration (2.15–2.8 μg/10 μl/per each CM) was assessed by Bradford protein assay. ATP (10 μM) was used as positive control for microglial cell recruitment. The migrating BV2 cells were detected by Hoechst staining and the number of cells was counted on dissected membranes transferred on glass slides and mounted with Vectashield mounting medium (Vector Laboratories, Inc. on LinkedIn). Three different counts were performed under a Nikon Eclipse Ti microscope with motorized stage.

### Immunofluorescence analysis of BV2 cells

For detection of CX3CR1 receptor, BV2 cells were grown on Poly-L-Ornithine-Coated Glass Coverslips in DMEM with 10% FBS, 1% P/S, for 24 h and then washed and treated with CM LSCI (1:3, CM LSCI:DMEM lacking FCS) or CM CTR, for 24 h BV2 cells were fixed with 4.0% paraformaldehyde, treated with 0.2% Triton X-100, and blocked with 2% normal goat serum (NGS, Sigma-Aldrich). Afterwards, they were sequentially incubated with anti-CX3CR1 antibody (1:100, Santa Cruz, USA), followed by FITC-conjugated goat anti-rabbit IgG, and 4,6-diamidino-2 phenylindole (DAPI, Sigma-Aldrich, Germany) solution, and examined using a fluorescence and confocal microscope (Leica, Germany).

### Western blot analyses

Western Blot analysis was carried out from BV2 cell extracts as previously described (Salzet et al., 1993). Blots were blocked with 5% milk in PBS for 45 min and incubated overnight at 4°C with a rabbit CX3CR1 antibody diluted 1/200. Detection was performed by enhanced chimioluminescence (Amersham, France) after 1h incubation with a peroxidase-conjugated secondary antibody (Aventis, Sanofi Pasteur, France) diluted at 1/20,000.

### Dorsal root ganglion explants

DRGs were isolated from the thoracic and lumbar spinal levels of a total of 3 Wistar rats (P2, *n* = 50). Under aseptic conditions, the DRG explants were prepared by trimming nerve roots using microsurgical scissors. Afterwards each explant was transferred to laminin pre-coated glass slides (0.1 mg/ml) in 12-well tissue culture plates (Costar, Corning, USA) with DMEM/F12 culture medium supplemented with: (i) CM CTR, (ii) CM RSCI segment, (iii) CM LSCI, (iv) CM CSCI (1:3, CM: DMEM) during 7days *in vitro* (7DIV). Control DRGs neurite outgrowth was induced with DMEM-F12 containing epidermal growth factor (EGF) and basic fibroblast growth factor (bFGF) (20 ng/ml/ each factor, 1% B27, 0.5% N2) during 7DIV. For all DRGs we have used standard IHC procedures to visualize neurite outgrowth. Briefly, DRGs were incubated in mouse anti-Neuron-specific class III beta-tubulin (TUJ1) (1:200; Merck) for 24 h at 4°C. Afterwards, they were washed in 0.1 M PBS and incubated for 2 h with goat anti-mouse IgG (Alexa Flour 488). For nuclear staining, we used DAPI (1:200). Finally, they were mounted, and cover slipped with Vectashield mounting medium (Vector Laboratories, Inc.). Digitized images of DRGs/per treatment (*n* = 6) were captured and saved with NIS-Elements Ar Microscope Imaging Software (Nikon). The outgrowth of neurite was analyzed at identical sampling fields, for each CM experiment by Image J software according to the above mentioned method applied for primary antibodies quantification.

### Statistical analysis

All data are reported as the mean ± s.e.m. One-Way ANOVA followed by the Tukey-Kramer test for multiple comparisons was used in the analyses of the Iba1+/CX3CR1+/CD206 in the spinal sections and for the chemotaxic results. Repeated-measures Two-Way ANOVA followed by the Tukey-Kramer test was used for the DRGs/TUJ1+ neurite outgrowth analysis. Statistical significance was set at ^*^p < 0.05; ^**^P < 0.01, ^***^P < 0.001.

### Proteomic studies

#### Digestion of CM

Fifty μL of the solution of CM obtained from the control spinal cord and along the lesion or rostral and caudal segments after SCI were added to 20 μL of a solution of DTT (50 mM) in NH_4_HCO_3_ buffer (50 mM) (pH = 8) and heated for 15 min at 95°C. After cooling, 20 μL of a solution of IAA (100 mM) in NH4HCO3 buffer (50 mM) were added and the mixture was incubated for 15 min at room temperature in the dark. 10 μL of a solution of trypsin (20 μg/mL) in NH_4_HCO_3_ (50 mM) were then added and the sample was incubated overnight at 37°C.

#### Tissue protein extraction

Twenty μm spinal cord tissue sections were mounted on a parafilm covered glass slide and the tissue was microdissected manually using a binocular. The pieces were extracted by incubating in 20 μL of 50 mM bicarbonate buffer containing 50mM DTT and 1% SDS at 55°C for 15 min. The extracts were then loaded on 12% polyacrylamide gel and separated at 70V for 15 min and then 120V until the dye front reaches the other end of the gel. After migration, the gel was incubated in the gel fixative solution for 30 min and stained with colloidal Comassie brilliant blue overnight. The stain was removed by washing the gel four times with distilled deionized water.

#### In gel digestion

The gel was cut into ten pieces. Pieces were washed with 300 μL of distilled deionized water for 15 min, 300 μL of ACN for 15 min and 300 μL of NH_4_HCO_3_ 100mM (pH8) for 15 min. Then a mix of 300 μL of NH_4_HCO_3_/ACN (1:1, v/v) for 15 min and 300 μL of ACN for 5 min. Band pieces were dryed in a Speedvac for 5 min. The reduction of cystine residues was made with 50 μL of 10 mM of DTT in NH_4_HCO_3_ 100 mM (pH8). Pieces were incubated at 56°C for 1 h. Alkylation of cystine was made with 50 μL of 50 mM of IAA in NH_4_HCO_3_ 100 mM (pH8). Pieces were incubated at room temperature in the dark for 30 min. Band pieces were washed a second time with 300 μL of NH_4_HCO_3_ 100 mM (pH8) for 15 min. Then a mix of 300 μL of NH_4_HCO_3_/ACN (1:1, v/v) for 15 min and 300 μL of ACN for 5 min. Band pieces were dryed in a Speedvac for 5min. A digestion of band pieces was made with trypsin (12.5 μg/mL) in NH_4_HCO_3_ 20 mM (pH8), enough to cover pieces. Pieces were incubated at 37°C overnight. Peptides were extracted on shaking platform with 50 μL of FA 1% two times for 20 min, then 150 μL of ACN for 10 min. The supernatant was transferred in new tube and dried with Speedvac.

#### NanoLC-HR-MS/MS

Samples were separated by online reversed-phase chromatography using a Thermo Scientific Proxeon Easy-nLC system equipped with a Proxeon trap column (100 μm ID × 2 cm, Thermo Scientific) and a C18 packed-tip column (100 μm ID × 15 cm, Nikkyo Technos Co. Ltd.). Peptides were separated using an increasing amount of acetonitrile (5–40% over 110 min) at a flow rate of 300 nL/min. The LC eluent was electrosprayed directly from the analytical column and a voltage of 1.7 kV was applied via the liquid junction of the nanospray source. The chromatography system was coupled to a Thermo Scientific Orbitrap Elite mass spectrometer programmed to acquire in a data-dependent mode. The survey scans were acquired in the Orbitrap mass analyzer operated at 120,000 (FWHM) resolving power. A mass range of 400–2000 m/z and a target of 1E6 ions were used for the survey scans. Precursors observed with an intensity over 500 counts were selected “on the fly” for ion trap collision-induced dissociation (CID) fragmentation with an isolation window of 2 amu and a normalized collision energy of 35%. A target of 5000 ions and a maximum injection time of 200 ms were used for CID MS^2^ spectra. The method was set to analyze the 20 most intense ions from the survey scan and dynamic exclusion was enabled for 20 s.

#### Data analyses

Tandem mass spectra were processed with Thermo Scientific Proteome Discoverer software version 1.3. Resultant spectra were searched against the Swiss-Prot® *Rattus norvergicus* database (version January 2012) using the SEQUEST® algorithm. The search was performed choosing trypsin as the enzyme with two missed cleavages allowed. Precursor mass tolerance was 10 ppm, and fragment mass tolerance was 0.5 Da. N-terminal acetylation, methionine oxidation and arginine deamination were set as variable modifications. Peptide validation was performed with the Percolator algorithm. Peptides were filtered based on a *q*-Value below 0.01, which corresponds to a false discovery rate (FDR) of 1%.

#### Label free quantification with scaffold 4 software

Scaffold (version Scaffold 4.0.6.1, Proteome Software Inc., Portland, OR) was used to validate MS/MS based peptide and protein identifications and label free quantification (Gstaiger and Aebersold, 2009). Peptide identifications were accepted if they could be established at greater than 50% probability by the Peptide Prophet algorithm (Keller et al., 2002) with Scaffold delta-mass correction. Protein identifications were accepted if they could be established at greater than 90% probability and contained at least 2 identified peptides. Protein probabilities were assigned by the Protein Prophet algorithm (Choi et al., 2008). Proteins that contained similar peptides and could not be differentiated based on MS/MS analysis alone were grouped to satisfy the principles of parsimony. Normalization was done on top 3 total ion current (TIC) in addition to spectral counting.

## Results

**Figure 1:**
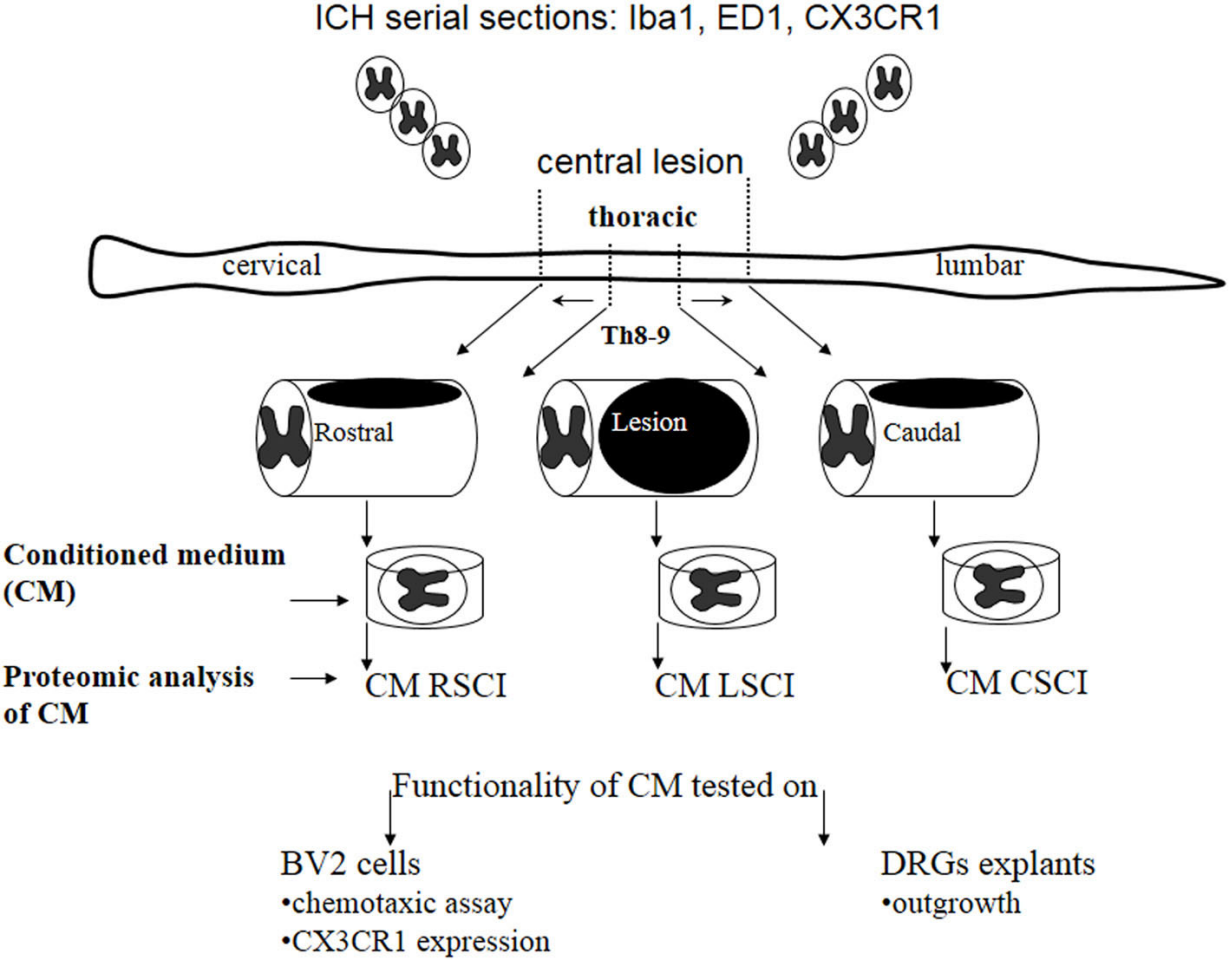
**Schematic design of the experimental procedure used in this study**.

Here, we investigated nature of the factors released in CM derived from three spinal cord segments (rostral, central lesion, caudal) obtained 3, 7, and 10 days after SCI and correlated its molecular composition with the *in vitro* and *in vivo* analyses (Figure 1).

### BV2 cells are stimulated with CM from injured spinal tissue

#### Microglia recruitment response to CM from SCI

**Figure 2:**
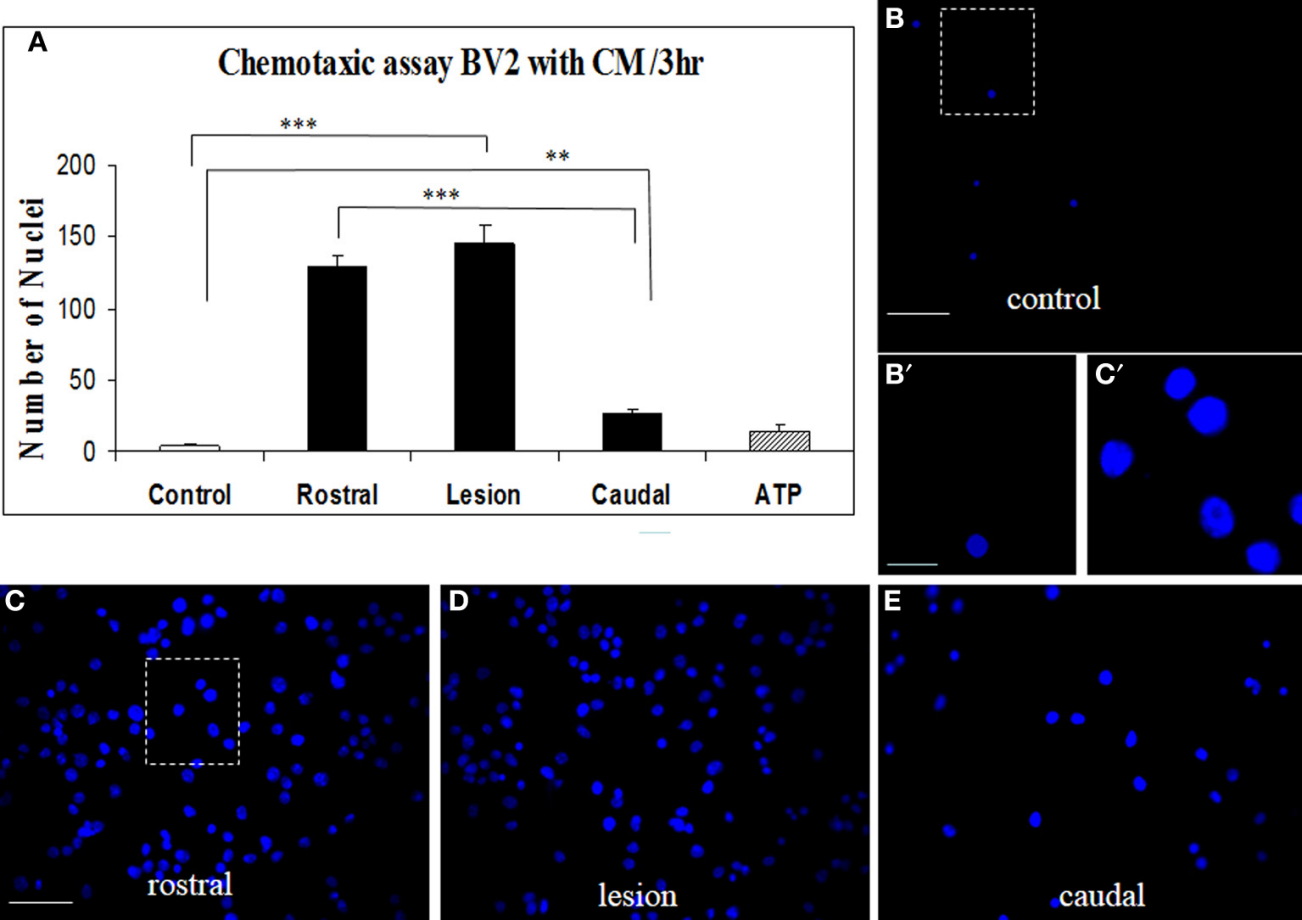
**CM SCI-induced BV2 cell chemotaxis in the transwell assay**. Activated BV2 cells were quantified by counting the number of Hoechst-labeled cells attached to the Boyden membrane after incubation with CM from control spinal segment **(A,B,B′)**, with CM from rostral **(A,C)**, lesion **(A,D)** and caudal **(A,E)** segments after SCI and ATP **(A)**. Significant increase of attached microglia occurred when BV2 cells were incubated with CM from rostral **(A,C)**, lesion **(A,D)** and caudal **(A,E)** segments. Note, marked nuclear hypertrophy of microglia following CM LSCI activation **(C′)** when compared to control **(B′)**. Data are represented as mean ± s.e.m. ^**^P < 0.01, ^***^P < 0.001, One-Way ANOVA followed by Tukey-Kramer test. Scale bars **(B–E)** = 50 μm; (**B′,C′)** = 10 μm

Proteins secreted from segmental fragments of injured and control spinal cords were analyzed after 24 h *in vitro* incubation. The regionalization between rostral and caudal segments from the lesion has been taken into account and each collected CM has been individually tested on microglial BV2 cells using Boyden chambers. BV2 cells were counted by using Hoechst-labeling (Figure 2A). More than a 37-fold increase of attached microglial cells were observed with the CM from the rostral and lesion regions (Figures 2A,C,D), compared to the CM from control or ATP application (number of nuclei in the identical sampling fields: control = 4.2 ± 1.6, rostral = 129.6 ± 8.5, lesion = 145 ± 12, caudal = 27.1 ± 3.8, ATP = 15.0 ± 5.3) (Figures 2A,B). In addition, comparison of microglia recruitment showed 5-fold higher activity using lesion and rostral CM when compared to the caudal one (Figures 2A,E). Furthermore, a marked nuclear hypertrophy of microglia was detected following activation, compared to control (Figures 2B′,C′).

### DRGs neurite outgrowth

**Figure 3:**
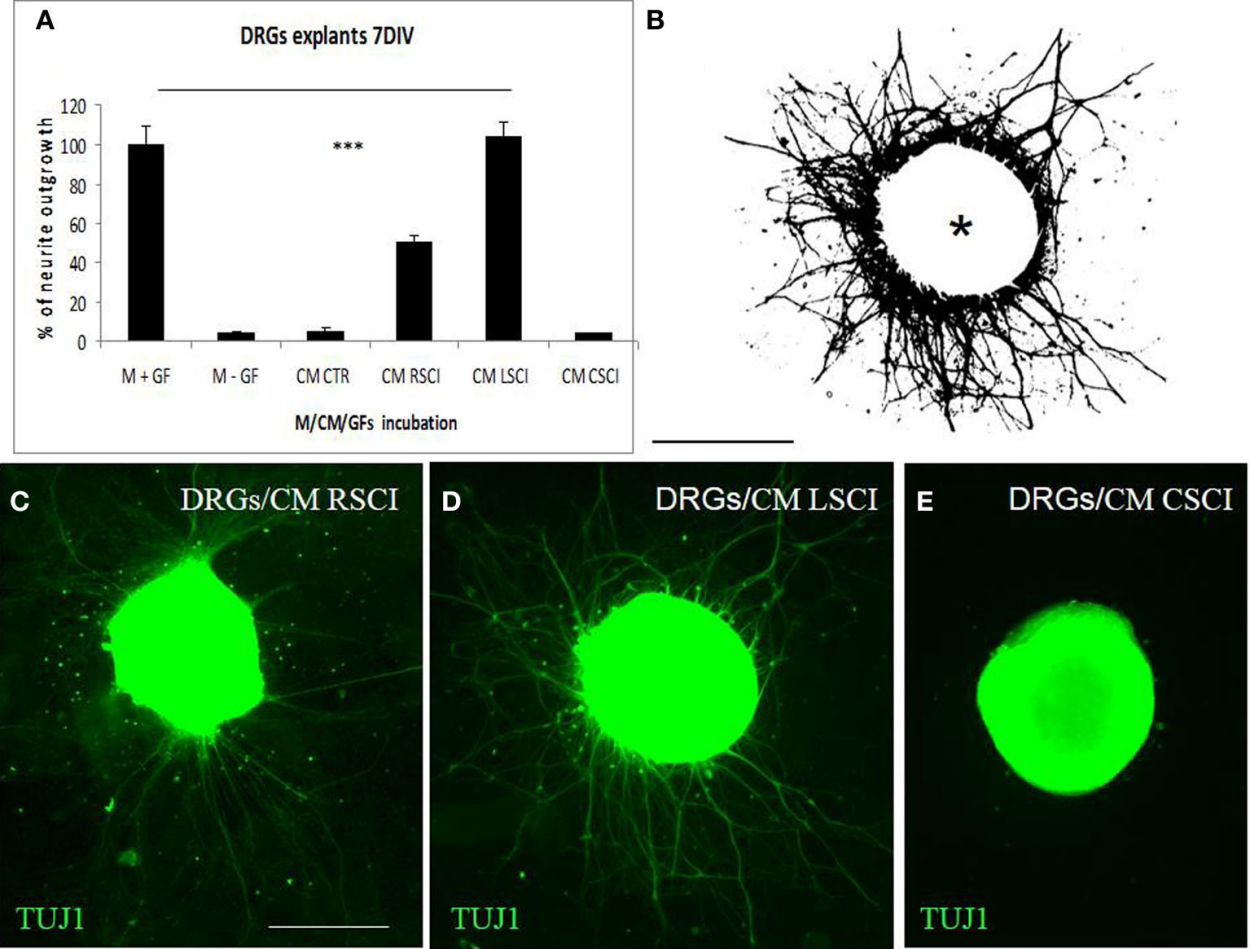
**Quantification of DRG explant neurite outgrowth in response to culture media**. M+GF (DMEM+ growth factors), M-GF (DMEM without growth factors), and conditioned media: CM CTR (control spinal cord), after SCI: CM RSCI (rostral segment), CM LSCI (lesioned segment), CM CSC (caudal segment) **(A)**. The image of TUJ1 stained DRG explants after selecting the area to be analyzed and thresholding the number of pixels covered by the extending neuritis, but not the DRG cell body (asterisk) **(B)**. Representative immunofluorescence images of TUJ1 positive DRG explants exposed to CM RSCI **(C)**, CM LSCI **(D)**, and CM CSC **(E)**. Scale bars **(B–E)** = 400 μm.

For quantification of neurite growth, the surface area outside the ganglion that was covered with neurites was determined (Figure 3B). This showed that enhanced TUJ1 positive neurite outgrowth from DRG explants was induced with CM LSCI (Figure 3D), and about half the growth was observed after culturing with CM RSCI (Figure 3C), if compared with control (Figure 3A). Conversely, almost no outgrowth from DRG explants was found after incubation with CM CSCI (Figure 3E), CM CTR nor with media lacking GFs (M-GFs) (Figure 3A). Thus, the outgrowth induced with CM LSCI was the most pronounced, and DRG explants sent large radial projections to the peripheral area (<500 μm). In addition, bright field images of DRGs confirmed a high number of migrating cells with neuron-like bipolar and fibroblast-like morphology scattered around those explants expressing high neurite outgrowth (CM CTR, CM LSCI) (Suppl. data 1). The mean value % of neurite outgrowth for each group was: 100% ± 9.1 = M + GFs; 4.07% ± 1.28 =M − GFs; 5.42% ± 1.7 = CM CTR; 50.38% ± 3.15 = CM RSCI; 103.9% ± 7.76 = CM LSCI; 4.03% ± 0.76 = CM CSCI. (^***^P < 0.001).

#### Proteomic studies

**Figure 4:**
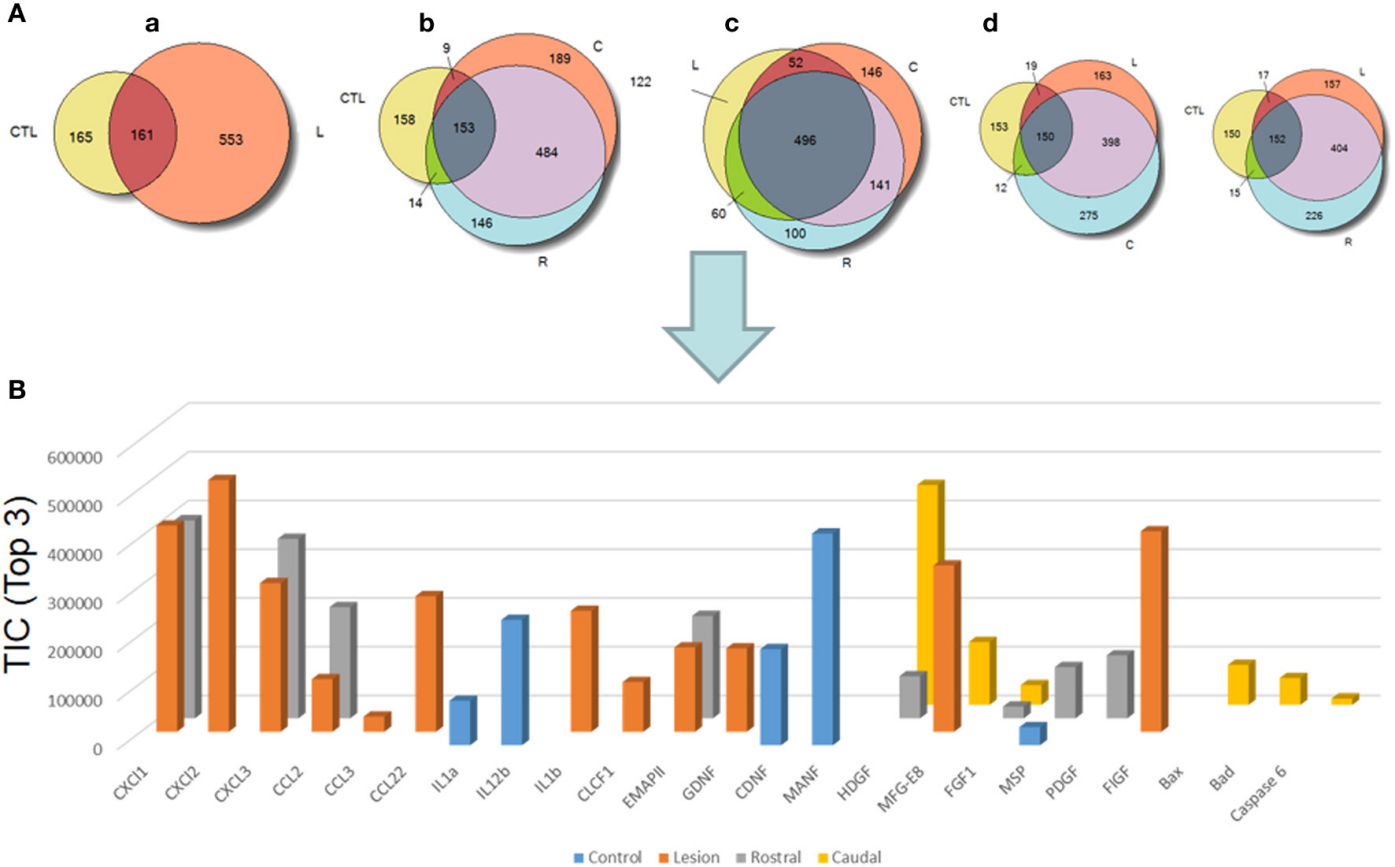
**(A)** Venn diagram representations of the common identifications after shot-gun analyses between CM derived from rostral, caudal, lesion SCI segments and control spinal cord segments without lesion. **(a)** consists of a comparison between control (CTL) and lesion (L), **(b)** Control (CTL), Rostral (R) and Caudal (C), **(c)** lesion, rostral, caudal and **(d)** control, lesion, rostral or control, lesion, caudal (see Suppl. data 1–4). **(B)** Label–free quantification of immune modulators, neurotrophic, and growth factors and apoptotic molecules identified in control, rostral, caudal, lesion segments using Scaffold_4.0.6.1. (CLCF1, cardiotrophin-like cytokine factor 1; EMAPII, endothelial monocyte-activating polypeptide; GDNF, glial cell-derived neurotrophic factor; CDNF, conserved dopamine neurotrophic factor; MANF, mesencephalic astrocyte-derived neurotrophic factor; HDGF, mesencephalic astrocyte-derived neurotrophic factor; MFG-E8, milk fat globule-EGF factor 8 protein; FGF, fibroblast growth factor; MSP, macrophage stimulating protein; PDGF, platelet-derived growth factor; FIGF, C-Fos induced growth factor).

We pointed out in our above results that a regionalization occurred between the rostral and the caudal segments in terms of factors secreted, i.e., neurotrophic and chemoattractant from the rostral segment, whereas none were from the caudal one. In this context, we investigated at a proteomic level the content of the SCI CM released from each spinal cord segment in order to identify these secreted factors. These experiments have been done on 4 SCI rats and 4 sham-operated rats per each survival (3, 7, 10 days). Among the identified proteins, those with a score under 5 were removed from subsequent analyses because they were identified from the MS/MS to have less than two peptides. We then evaluated the number of common peptides between the CM of interest. Each accession number, protein description, gene name and relative score associated with the selected proteins is reported in (Suppl. data 2–4). The Venn diagram presented in Figure 4A illustrates the common identifications between rostral, caudal, lesion SCI and control without lesion. Specific proteins have also been detected for each segment after SCI. In fact, 553 specific proteins have been detected in lesion (Figure 4A). 153 ± 3 (*n* = 5) proteins are in common between control, rostral and caudal SCI. In addition, 14 ± 1 (*n* = 5) proteins are in common between control and rostral SCI whereas only 9 ± 4 (*n* = 5) are found in both caudal SCI and control (Figure 4Aa–d). Comparison between caudal and rostral SCI showed 481± 3 (*n* = 5) proteins in common. Experiments have been redone 5 times and the number of proteins identified in each condition was very close (±4). The Venn diagram (between rostral and caudal SCI after elimination of the proteins in common with the ones detected in normal SC) confirmed the chemotaxis data. In fact, not only do the rostral and caudal CM have proteins in common but also some specific proteins compared to each other (Figure 4A). As presented in Table 1, for the first time in shot-gun analyses from CM, chemokines have been identified and quantified by label–free quantification (Suppl. data 5) in the lesion and rostral segments, e.g., CXCL1; CXCL2; CXCL7, CCL2, CCL3, CCL22, CLCF1, EMAP II. These chemokines and the neurotrophic factors (TGF®, FGF-1, PDGF, and FIGF1) detected in these regions clearly showed the presence of factors that are known to be immune-modulators and are neurotrophic (Figure 4B). Together, these factors are known to polarize the macrophages/microglial cells in the M2 phenotype (Figure 4B). Thus, three days after SCI, factors secreted by the cells present at the lesion and in the rostral segment (Table 1) are in neuroprotective and neurotrophic environment whereas those from the caudal region are more apoptotic. The complete screening of the CM confirmed such evidence. In fact, more neurotrophic factors have been detected in the lesion and rostral part, i.e., CTGF (Connective tissue growth factor), NOV (Protein NOV homolog), PIGF (Placenta growth factor), FGF-1 (Fibroblast growth factor 1), BMP 2 or BMP3 (Bone morphogenetic proteins (2 or 3), NGF, PGF, TGF beta (1–3) (Transforming growth factor beta), periostin, GAP-43, neurotrimin, neurofascin, Hepatocyte growth factor-regulated tyrosine kinase substrate (HGS). Also, molecules involved in neuronal development/differentiation/neuronal migration, i.e., CRIP1 (Cysteine-rich protein 1), DRP-5 (Dihydropyrimidinase-related protein 5), Negr1 (Neuronal growth regulator 1), NCAN (Neurocan core protein), CD44, Wnt8, syndecan-4, nexin, Bcl-2, were identified. Specific factors involved in immune cell chemotaxis or cellular adhesion, including complement factors (C1qb, C1qc, factor D, factor I, CD59), tetraspanins (CD9, CD82), and CD14 have also been characterized. In the contrast, proteins from the caudal region are more related to necrosis factors (BAX, BAD, Casapase 6, neogenin), cytoskeleton proteins, synaptic vesicle exocytosis, chemoatractant factors and neuronal postsynaptic density (Figure 4B, Table 1). Only BMP2, BMP3, and HGS have been detected. Comparison of proteins identified from the lesion rostral and caudal regions gave 122 proteins specific to the lesion site, 100 to the rostral segment, and 146 to the caudal one (Table 1). Nevertheless, 60 proteins are in common between the rostral and the lesion, and among these proteins, chemokines and neurotrophic factors have been only detected in these segments and never in the caudal part. Thus, 3 days after SCI, a clear regionalization occurs between the rostral and caudal regions (Table 1). These data can explain why initial sprouting and neurite outgrowth occur within the rostral and lesion parts and no regenerative processes can be found in the caudal part, although microglial cells are present and are in an activated state. Tissue proteomic analyses have been performed 3, 7, and 10 days after SCI. 801 common proteins to both 3 conditions have been identified. 58 specific proteins have been identified at 3 days, 446 at 7 days, 62 at 10 days after SCI. 230 proteins are common to both 7 and 10 days after SCI whereas only 41 between 3 and 10 days after SCI. Neurotrophic factors have been identified at 3 days, diminished at 7 days and disappeared at 10 days after SCI. However 10 days after SCI, proteins related to synaptogenesis have been detected, e.g., R–SNARE synaptobrevine, Q–SNAREs (SNAP25), GTPases (Rab proteins family), les synaptogenines, syntaxines, synaptotagmine. This reflected that 10 days after SCI proteins involved in axonal reconnection and synaptic transmission are expressed. This reflects that a neurorepair process has started (Table 2). In contrast, at the caudal segment, the protein profile is always inflammatory and apoptotic whatever the days after SCI (Data not shown).

**Table 1:**
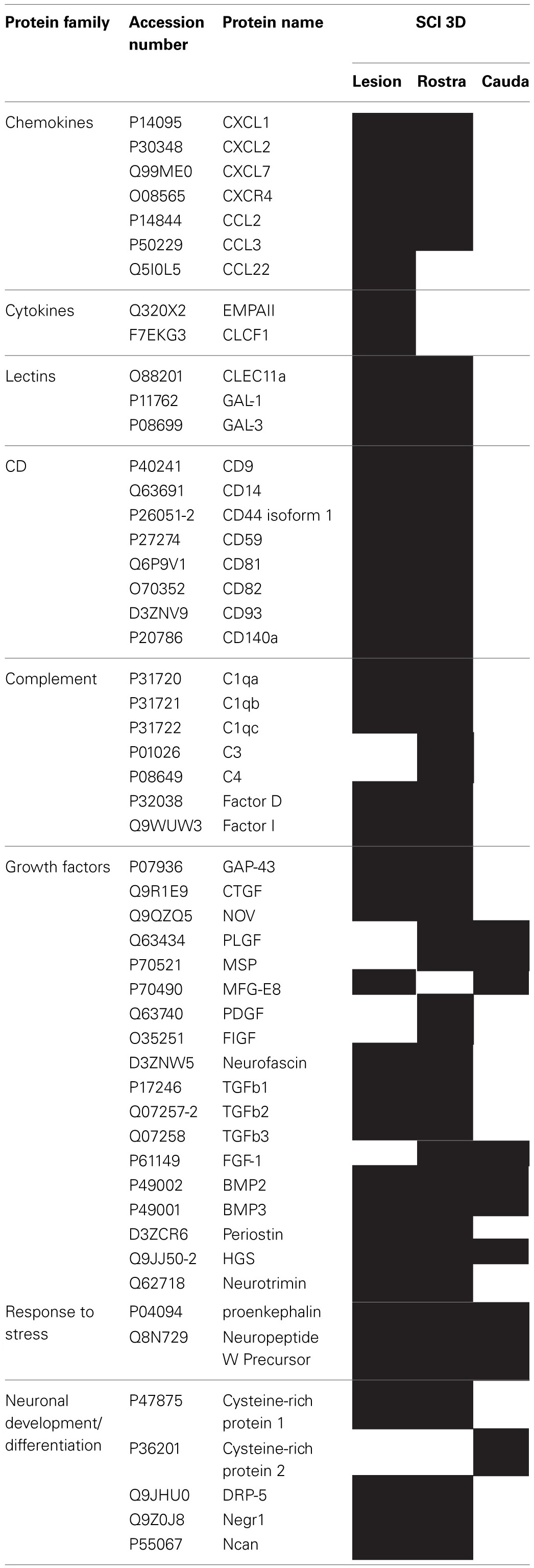
**List of proteins identified by shot-gun issue from CM (24 h) of central lesion, adjacent rostral and caudal segments after SCI at 3 days**.

*The black shade corresponds to the proteins identified in the concerned case*.

**Table 2:**
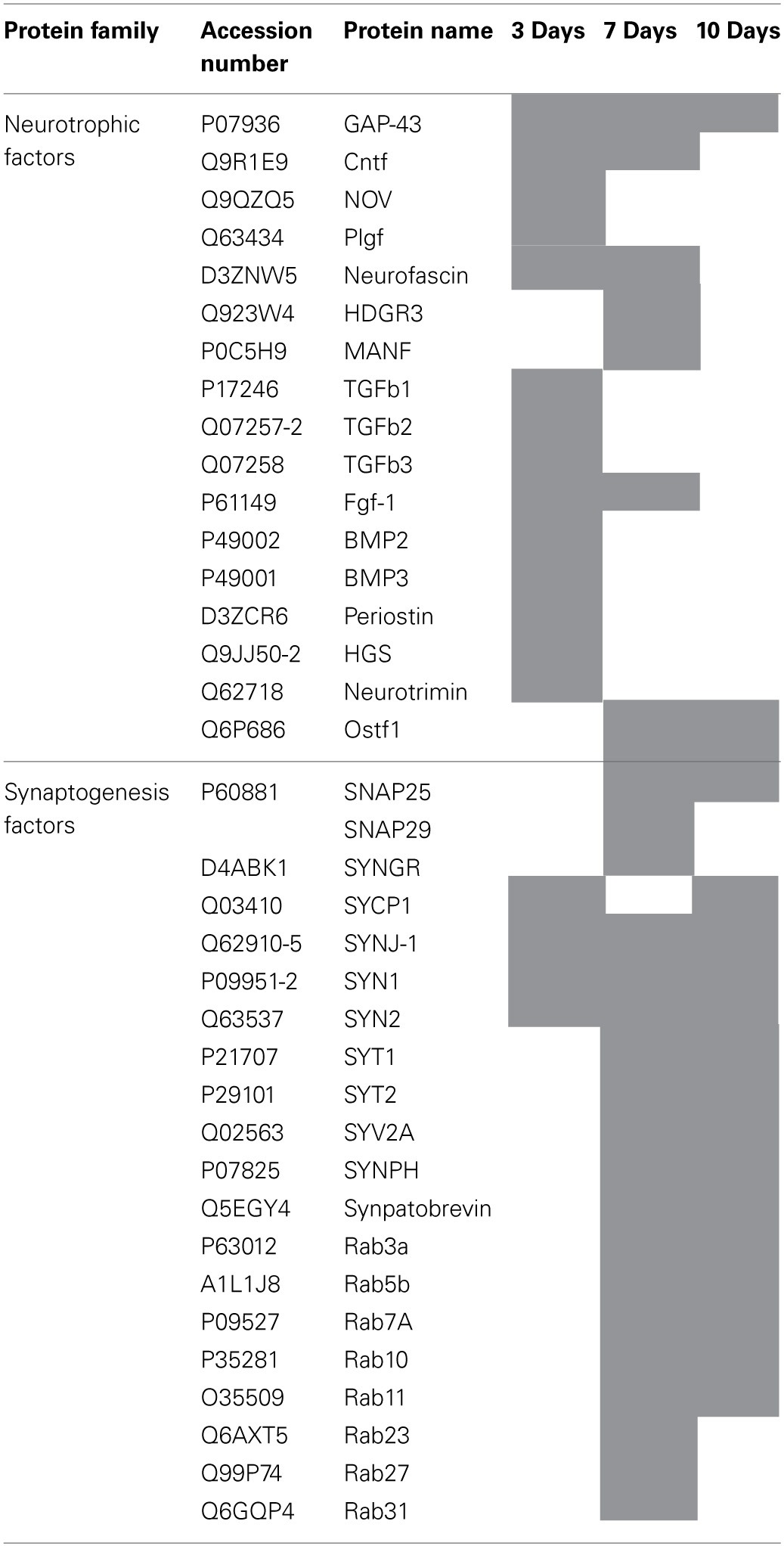
**Identified proteins from rostral tissue sections in time course after SCI**.

*The gray shade corresponds to the proteins identified in the concerned case*.

### Microglia expression in control and injured spinal cord tissue

**Figure 5:**
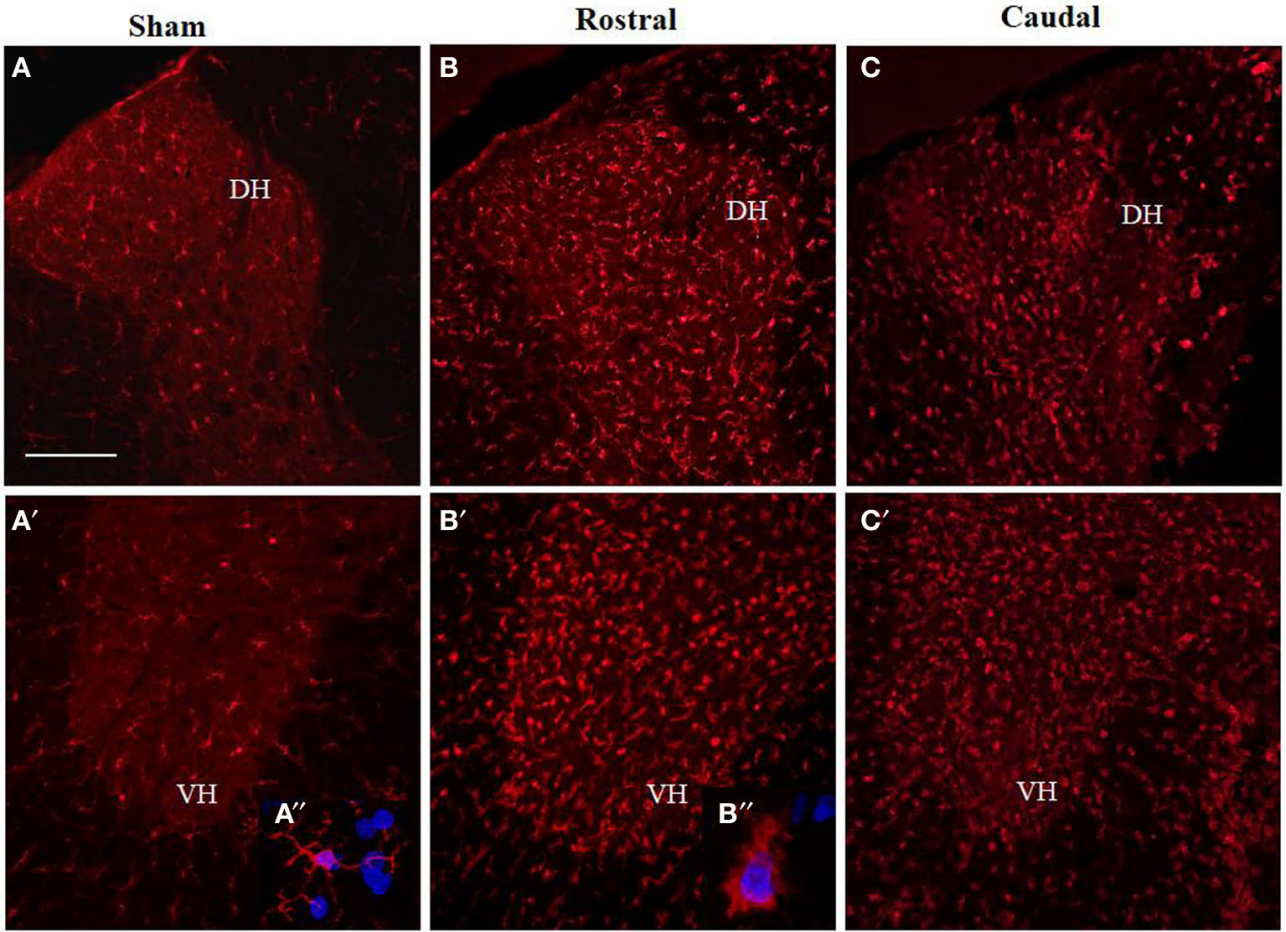
**Comparison of Iba1 immunoreactivity (IR) in coronal thoracic spinal cord sections of sham rats (A,A′) and rats 3 days after injury in rostral (B,B′) and caudal segments (C,C′) in the dorsal (upper) and ventral horn (lower panel)**. Note, significantly increased number of activated Iba1-positive microglia in both rostral and caudal segments within the dorso-ventral axis of gray matter as well as throughout corresponding white matter. Scale bars **(A,A′–C,C′)** = 150 μm.

**Figure 6:**
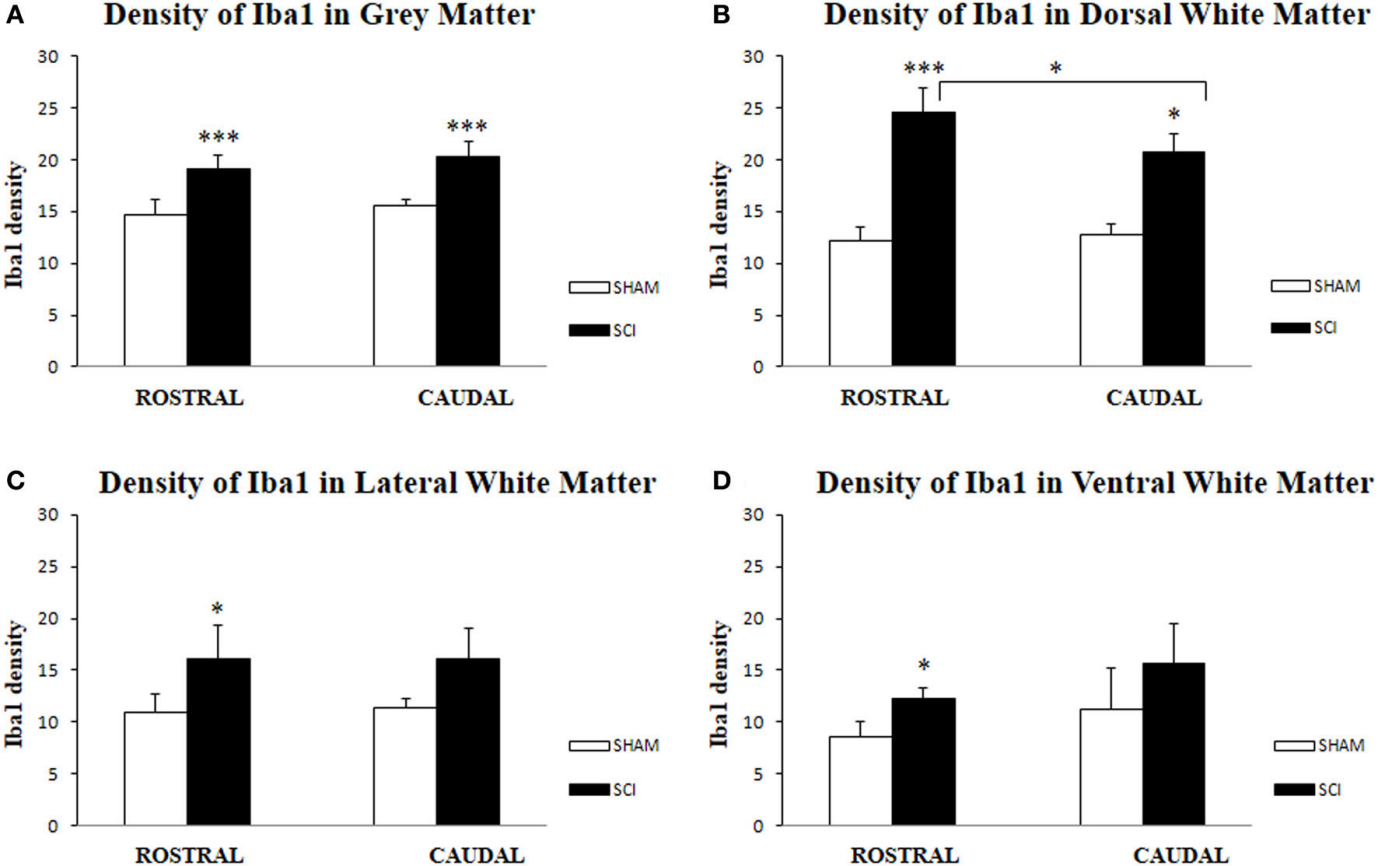
**Density of Iba1-IR microglia (*n* = 4) in the spinal cord of sham and rats after SCI in gray (A), White Matter (B,C,D); Dorsal White Matter (B), Lateral White Matter (C) and Ventral White Matter (D)**. Density of Iba1-IR positive microglia was significantly higher in all analyzed areas after SCI when compared to sham. Significant differences between rostral and caudal segments occurred only at the dorsal white matter (B). Data are presented as mean ± s.e.m., ^*^P < 0.05 **(B–D)**, ^***^P < 0.001 **(A,B)** for sham vs. SCI, or rostral to caudal after SCI for group comparison (ANOVA) followed by Tukey-Kramer test.

Antibodies against Iba1 exhibited moderate expression of resident microglia (Figures 5A,A′,A″). After SCI, microglia changed into an activated phenotype: marked cellular hypertrophy and thick, short and radially projecting processes with fewer ramifications (Figures 5B,B′,B″,C,C′). This was confirmed by quantification analyses, which showed some disparities between microglia accumulated in the dorsal white matter of the rostral and caudal spinal cord segments (Figures 5C,C′, 6A–D). Significant enhancement of Iba1 positive microglia occurred in the rostral dorsal white matter (Figure 6B), while in other areas we did not detect significant differences between the rostral and caudal segments. The spinal cord tissue at the central lesion was severely damaged, losing its typical anatomical architecture (Suppl. data 6). Therefore, we could not quantify Iba1 positive profiles at the lesion site. In addition, the entire dissected central segment (±5.0 mm) showed dense and homogenous infiltration of Iba1 and ED1 positive macrophages (Suppl. data 7). However, out of the central lesion, toward the rostral or caudal segments, Iba1 macrophages together with migrated ED1 monocytes occupy mainly the lesion cavity and accumulate at the lesion penumbra (Suppl. data 7). In addition, we could clearly distinguish the monocyte-derived amoeboid Iba1 microglia concentrated within the dorsal WM, while hypertrophied microglia with ramified processes retained at the gray matter tissue (Suppl. data 7). Furthermore, by immunofluorescent double labeling of ED1 and Iba1 we confirm the distribution of macrophages throughout the rostro-caudal extent of spinal contusion lesions (Suppl. data 7). All data are included in Table 3. To confirm the key role of inflammation and not gliosis at 3 days after SCI we studied response of astrocytes by GFAP IHC. Here we show that injury resulted into decreased number of GFAP positive profiles, with short processes surrounded by debris. These GFAP+ profiles were impaired, when compared to fine star like astrocyte morphology found in sham spinal cord sections (Suppl. data 8).

**Table 3:** **Quantification of Iba1, CX3CR1 and CD206 immunoreactivities in sham, SCI tissue from rostral and caudal segments at 3 days**.

	**Sham rostral**	**SCI rostral**	**Sham caudal**	**SCI caudal**
**DENSITY OF Iba1**				
GM	14.6 ± 1.5	9.1 ± 1.3	15.5 ± 0.4	20.3 ± 1.4
WMD	12.1 ± 1.4	24.6 ± 6.2	12.8 ± 0.9	20.7 ± 3.7
WML	10.8 ± 1.7	16.1 ± 6.4	11.3 ± 0.8	16.1 ± 3.9
WMV	8.6 ± 1.4	12.2 ± 1.3	11.2 ± 4.6	15.6 ± 4.8
**DENSITY OF CX3CR1**				
GM	5.1 ± 1.07	22.8 ± 3.2	4.2 ± 1.8	16.2 ± 3.8
WM	2.0 ± 1.32	17.27 ± 4.4	3.0 ± 1.6	8.0 ± 2.6
**DENSITY OF CD206**				
GM	8.8 ± 1.1.7	27.8 ± 4.6	9.2 ± 1.9	28.2 ± 2.8
WM	4.8 ± 1.2.1	28.4 ± 5.4	5.4 ± 2.4	26.8 ± 4.4

### Expression of CX3CR1 receptor and CD206+ profiles along the rostro-caudal axis in spinal cord tissue

**Figure 7:**
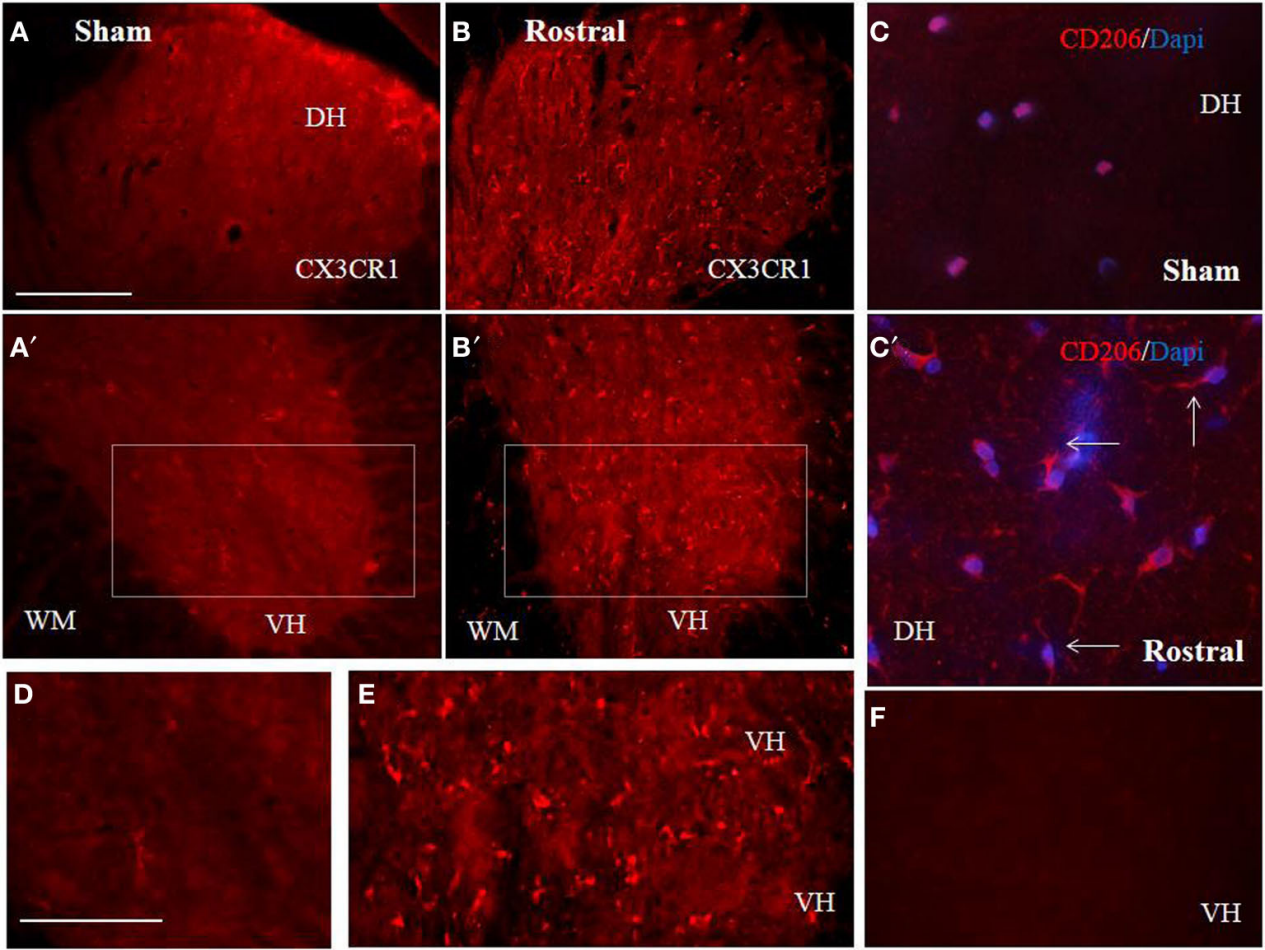
**Comparison of CX3CR1 expression in representative cross sections of thoracic spinal cord from sham and SCI rats at the rostral and caudal segmental levels**. CX3CR1 showed low expression pattern in sham, throughout GM (dorso-ventral axis) and WM **(A,A′,D)**, while injury stimulated CX3CR1 up-regulation **(B–E)**, particularly at rostral segments **(B,B′,E)**. Note, higher magnification of homogenous-membranous pattern of CX3CR1 in sham **(D)**, from **A′** = boxed area, or vesicular-dot-like patter of CX3CR1 after injury, rostral segment **(E)**, from **B′** = boxed area. Scale bars **(A–C′)** = 100 μm, **(D,E)** = 50 μm. A representative profile of CD206 (*n* = 4) expression in rostral spinal cord sections from sham **(C)** and SCI rats. Note, CD206+ macrophage like profiles with ramified and elongated processes after SCI. In the negative control no CX3CR1 or CD206+ expression occurred **(F)**. Scale bars **(A–C′)** = 100 μm, **(D,E)** = 50 μm.

**Figure 8:**
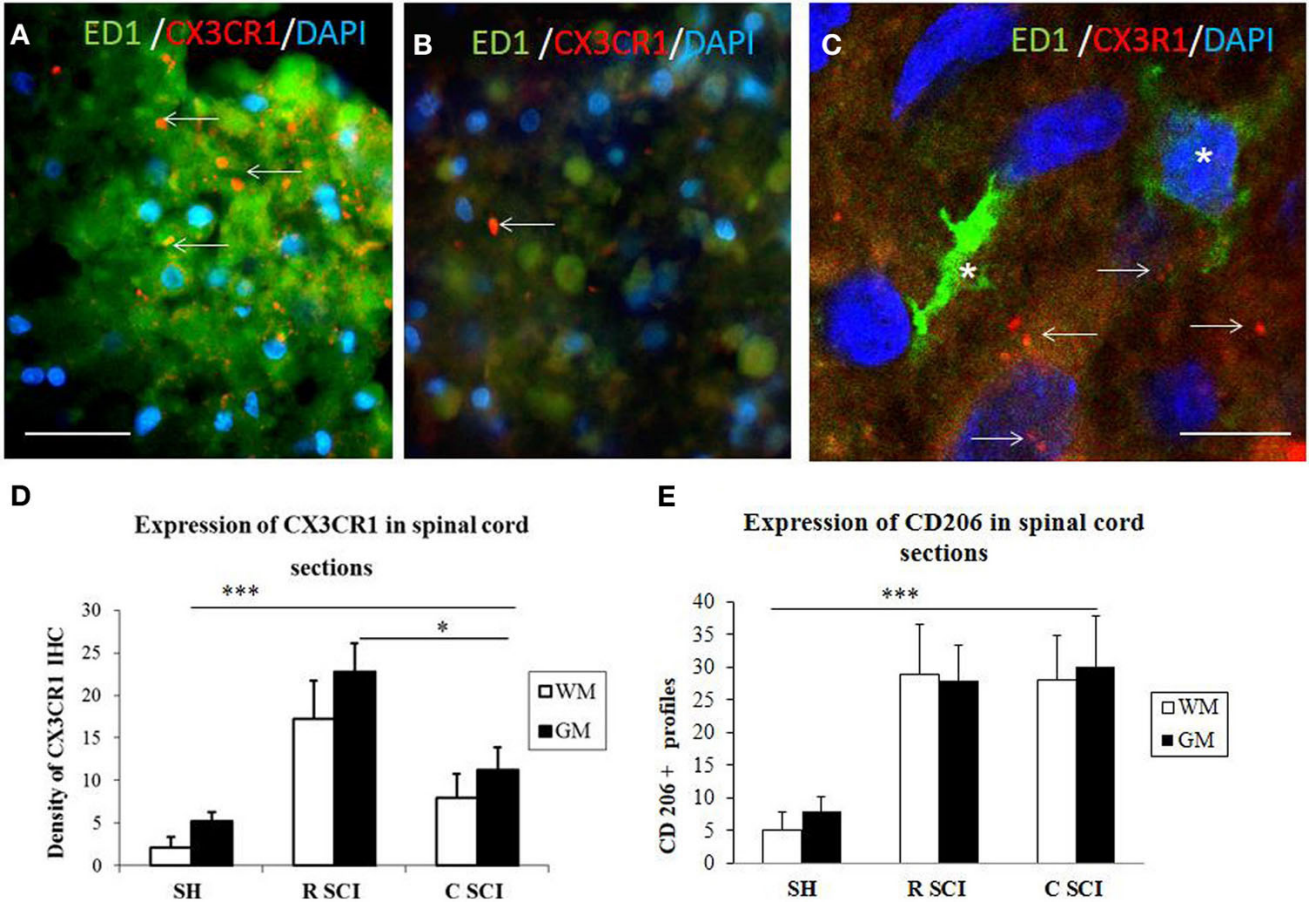
**Cross sections through dorsal white mater at the level of rostral (A) and caudal (B) segments of the injured spinal cord stained with CX3CR1 (red) and ED1 (green) antibodies revealing increased expression of CX3CR1 (red) in rostral segment compared to caudal (arrows)**. Note, no co-localization between CX3CR1 and ED1 labeled macrophages (see arrows indicated red dot for CX3CR1 labeling) **(C**, confocal image**)**. Density of CX3CR1+ (*n* = 4) and expression of CD206 profiles (*n* = 4) in the spinal cord sections of sham and 3 days after SCI in White /Gray Matter **(D,E)**. **(D)** Density of CX3CR1was significantly higher when comparing Sham (SH) sections with sections from injured spinal cord (SCI), sections from rostral (R SCI) compared with caudal (C SCI) segment within WM/GM. **(E)** CD206+profiles significaly increased when compared Sham (SH) sections with sections from injured spinal cord (SCI), not between R SCI and C SCI sections. Data are presented as mean ± s.e.m., ^*^P < 0.05, ^***^P < 0.001 for sham vs. SCI, or rostral to caudal after SCI for group comparison W/G matter (ANOVA) followed by Tukey-Kramer test. Scale bars **(A,B)** = 50 μm, **(C)** = 20 μm.

The proteomic data showed that factors released from the rostral and lesion segments express neuroprotective and immunomodulatory properties, whereas the ones found in the caudal are more inflammatory. In this context, we focus our attention on the CX3CR1 receptor which is known to be expressed in immune cells expressing an M2 profile characterized by production of neurotrophic factors and immune modulators like CXCL1, CXCL2, CXCL3, CCL1, CCL2, CCL22, EMAP II, and CLCF1, factors that were identified in CM derived from rostral and lesion segments. Expression of CX3CR1 in sham spinal cord tissue was low, with homogenous distribution in whole layers of the spinal cord with occasionally labeled microglia (Figures 7A,A′,C,D). However, SCI after 3 days induced a significant upregulation of CX3CR1 expression in the dorsal, ventral gray and white matter. This upregulation seems to co-localize with spinal microglia and recruited monocytes within damaged tissue (Figures 7B,B′,E). Higher expression of CX3CR1 was detected in rostral segments (Figures 7B,B′,E) when compared to the caudal spinal cord tissue at day 3 post-injury which was validated by quantification of CX3CR1 immunohistochemistry in coronal sections (Figure 8D). Similarly, CD206+ macrophages significantly increased in both rostral and caudal spinal segments when compared to corresponding areas of sham operated rats. However, we did not found differences along the rostro-caudal axis, thus the white and gray matter were infiltrated with large multipolar macrophages with elongated processes (Figures 7C,C′, 8E).

In order to evaluate the part of infiltrating macrophage that can also express the CX3CR1 receptor, double labeling using anti-CX3CR1 (red) and anti-ED1 (green) was performed in the dorsal white matter at the level of rostral and caudal segments three days after SCI. Results confirm that a clear labeling for CX3CR1 is found in the rostral segment (Figure 8A) and lower in the caudal segment (Figure 8B). No co-localization was detected between CX3CR1 and ED1 labeling indicating that macrophages did not express CX3CR1 receptors (Figure 8C, arrows indicate the Iba1 labeling distinct from the ED1).

### Modulation of CX3CR1 in BV2 cells

**Figure 9:**
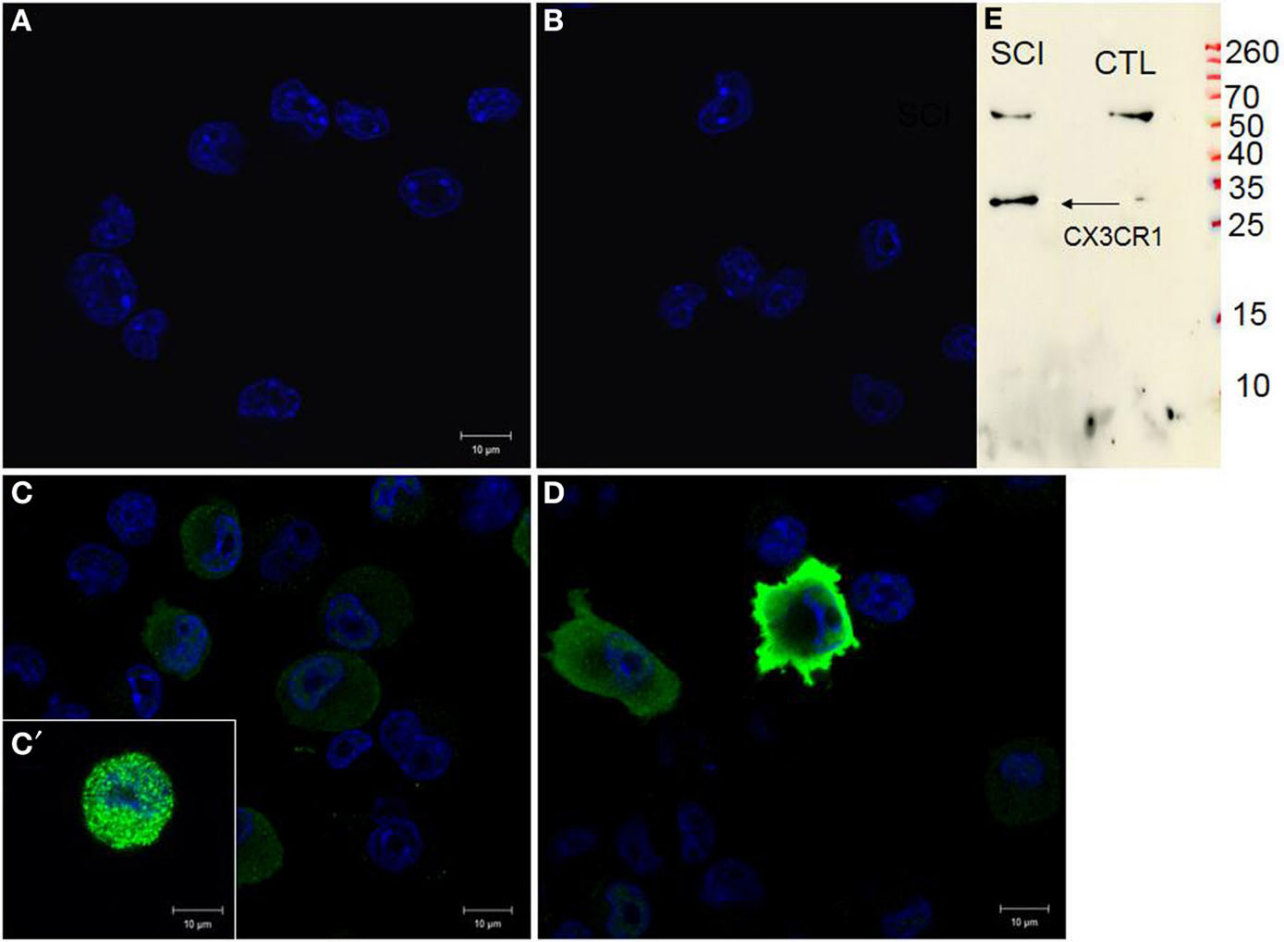
**CX3CR1 expression in control BV2 cells (A), stimulated with CM LSCI (C)**. Spindle shaped, multipolar or round BV2 cells following CM LSCI stimulation expressed intense CX3CR1 IR **(C,D)**. Scale bars **(A–D)** = 50 μm. Control **(B)**, only secondary antibody. Inset picture corresponds to Western blot analyses using anti-CX3CR1 from control or activated BV2 with CM LSCI **(E)**. Note, the band at 34 kDa correspond to the CX3CR1 and the band at 60 kDa is an unspecific band.

Naive BV2 cells did not show any signal for CX3CR1 (Figure 9A) similar to the negative control, when using only the secondary antibody (Figure 9B). Following 24 h stimulation of BV2 cells with CM RSCI, multipolar or round cells (arrows) expressed strong CX3CR1 immunofluorescence (Figures 9C,C′,D). Interestingly, it can be noticed that the labeling was either vesicular (Figure 9C′), or membranous (Figure 9D), which demonstrates an receptor from Golgi to membrane after activation of the BV2 cells with CM from an injured spinal cord. No labeling using anti-CKR2, a marker for M1 polarization of macrophages and microglial cells, has been found in control nor in BV2 cells stimulated with CM R SCI (data not shown). Western blot analysis showed the presence of a specific band at 34 kDa (Figure 9E) corresponding to the CX3CR1 receptor only in BV2 cells stimulated with CM RSCI.

## Discussion

In the present study, we show that the activated Iba1 positive microglia increased in all evaluated areas following SCI, but the significant differences between rostral and caudal segments occurred only within the dorsal white matter, where the impact of damage is most prominent in this SCI animal model. Thus, this discrepancy could be accounted in part to the presence or absence of macrophages derived from CNS-residing microglia or from blood-derived monocytes invading the most damaged regions at the rostral and caudal spinal levels (Mawhinney et al., 2012). Previous work have shown that after SCI, pro-inflammatory cytokines are upregulated by microglia and macrophages in the first few days after injury. These inflammatory mediators are also produced by other cell types in the lesioned spinal cord (Pineau and Lacroix, 2007; Pineau et al., 2010). Thus, after SCI, a complex array of chemokines and cytokines regulate myelopoiesis and intraspinal trafficking of myeloid cells. As these cells accumulate in the injured spinal cord, the collective actions of diverse cues in the lesion environment help to create an inflammatory response marked by tremendous phenotypic and functional heterogeneity. Indeed, it is difficult to attribute specific reparative or injurious functions to one or more myeloid cells because of convergence of cell function and difficulties in using specific molecular markers to distinguish between subsets of myeloid cell populations (Hawthorne and Popovich, 2011). The temporal activation of the myeloid cell lineage leads within minutes of SCI to the creation of a heterogeneous network of multifunctional cells that could be able of promoting injury and repair of neural tissue. Microglia are among the first myeloid cells to be set in motion, responding to changes in extracellular ions and ATP within minutes to hours post-injury followed by neutrophils (Hawthorne and Popovich, 2011). Neutrophils arrive first between 3 and 24 h post-injury. Then monocytes arrive later, ~2 to 3 days post-injury with maximal infiltration occurring 1–2 weeks post-injury. At 3 days post-injury data obtained by cDNA microarray and quantitative real-time PCR analyses, showed that macrophage expressed M1 and M2 markers, but the M1/M2 ratio determines whether CNS macrophages contribute to axonal regeneration after SCI (David and Kroner, 2011). Here we also confirm that CX3CR1 immunoreactivity was up-regulated after SCI, revealing most prominent expression within the gray and white matter of rostral spinal segments. This may coincide with both activated intraspinal microglia as well as with monocytes invading the lesion already 3 days post-injury, as confirmed in the present study with ED1 immunoreactivity. It is important to point out that monocyte recruitment after SCI is a dynamic process that initiates within the first days post injury, but is further accelerated during longer survival (Kullberg et al., 2001; Stanislaus et al., 2001). However, in the present study, co-localization experiments between CX3CR1 and ED1 did not confirm that macrophage expressed an M2 profile, while it seems more likely to be expressed in resident microglia.

Based on advanced proteomic analyses, we were trying to find a correlation between the immune response along the rostro-caudal axis and the content of released molecules 3, 7, and 10 days after injury. This is the first time that chemokines were identified by shot-gun analysis in the CM derived from rostral to caudal spinal cord segments. In particular, the immune factors (CCL2, CCL3, CCL22, CXCL1, CXCL2, CXCL7, EMPAII, CLCF1) that were detected in the CM from rostral segments with the neurotrophic factors (CTGF, NOV, PIGF, FGF-1 BMP 2, BMP3, NGF, PGF, TGF beta (1–3) periostin, GAP-43, neurotrimin, HGS) favor differentiation of microglia cells toward the M2 rather than the M1 phenotype. These data are in line with our findings showing increased CX3CR1 expression in rostral spinal segments as well as with previous data of M2 microglia polarization at the central lesion 3 days after SCI (Kigerl et al., 2009). In contrast to CX3CR1, the CD206+ profiles revealed similar response at both rostral and caudal segments. Furthermore, *in vitro* chemotaxis assays confirmed that BV2 cells were highly responsive to the cytokine cocktail present in the CM from lesion and rostral sites. Interestingly, the BV2 migratory potency induced by CM derived from rostral and lesion segments was 37-fold higher compared to the ATP or LPS stimulations that increase their migration by close to 3-fold due to the specific factors found in the complex CM (Rahmat et al., 2013). Our immunocytochemical studies prove that activated BV2 cells exposed to CM from the rostral segment over-expressed the CX3CR1 receptor, and this overexpression is known to correspond with the M2 profile. This was strengthened by western blot analysis and lack of labeling with C2KR, an M1 receptor. These data together with *in vivo* CX3CR1 expression are in close coherence with published transcriptomic experiments showing that in the injured spinal cord M2 gene expression is transiently expressed during 7 days after injury, while the M1 gene expression is maintained for up to 1 month (Kigerl et al., 2009). Moreover, a direct role of CSPG in controlling microglial and macrophage behavior after SCI was demonstrated. It seems that beneficial role of CSPG during the acute stage (regulating their phagocytosis or neurotrophic factor secretion) and its deleterious effect at later stages emphasizes the need to retain the endogenous potential of this molecule for recovery by controlling its levels at different stages of post-injury repair (Rolls et al., 2008).

Our tissue proteomic data also confirm this point. In fact, a temporal-spatial analysis in tissue proteomic has been undertaken at the rostral and caudal segment levels, 3, 7, and 10 days after SCI. Results shown that the proteome pattern is modified in course of time in the rostral segment whereas the caudal segment present the same pattern whatever the time after SCI. Neutrophic factors are found at 3 and 7 days after lesion and disappeared at 10 days. They are replaced by synaptogenesis factors reflecting the fact that a neurorepair process is taking place after 10 days SCI at the level of the rostral segment. These data are in line with the DRG experiments and our previous *in vivo* results demonstrating that neurite outgrowth takes place from rostral to lesion but never from caudal to lesion (Novotna et al., 2011) Furthermore, the content of chemokines, lectins, and growth factors in the rostral but not in the caudal segment clearly document the immediate inflammatory response together with activity-dependent factors released by neurons and glia.

In order to investigate the neurotrophic role of CM derived from lesioned tissue, we have also undertaken studies of neurite outgrowth in rat DRG explants. Our data validate that pronounced neurite sprouting of DRGs facilitated by CM from rostral and lesion segments are most likely mediated by the content of neurotrophic factors, i.e., FGF-1, NGF, PGF, BMP 2 or BMP3, GAP-43, neurotrimin, neurofascin, and other molecules involved in neuronal development/differentiation/ migration. Although the principal role of NGF/TrkA pathways in sensory axon outgrowth have been widely demonstrated, other neurotrophic factors including the BMPs (members of the TGFβ superfamily) or GAP-43 have to be taken into account. Particularly, the Smad1-dependent BMP signaling is developmentally regulated and governs axonal growth in the dorsal root ganglion neurons. Similarly, GAP-43, a membrane-bound protein, is expressed in neurons during axonal outgrowth development and is significantly up-regulated in DRGs during regeneration (Tsai et al., 2007; Parikh et al., 2011).

Taken together, these data could have a clear impact in clinics. It will be more important to stimulate neurite sprouting at the caudal region by inhibiting inflammation and polarization of M1 cells to the M2 state. These results demonstrate that each segment of the lesion has to be taken into consideration independently from each other in order to modulate inflammation, stimulate neurite outgrowth and functional reconnection.

### Conflict of interest statement

The authors declare that the research was conducted in the absence of any commercial or financial relationships that could be construed as a potential conflict of interest.
