# Supplementary material for: Alterations of protein composition along the rostro-caudal axis after spinal cord injury: proteomic, in vitro and in vivo analyses
Source: Front Cell Neurosci. 2014 Apr 17;8:105. doi: 10.3389/fncel.2014.00105 (PMC4028999; doi:10.3389/fncel.2014.00105)
Supplement: Supplementary file 1 [file Presentation1.ZIP › Supplementary Material Legends.docx]

***Supplementary Material***

**Alterations of protein composition along the rostro-caudal axis after spinal cord injury: proteomic, *in vitro* and *in vivo* analyses**

**Dasa Cizkova^1,2*^, Françoise Le Marrec-Croq^1*^, Julien Franck^1^, Lucia Slovinska^2^, Ivana Grulova^2^, Stéphanie Devaux^1^, Christophe Lefebvre^1^, Isabelle Fournier^1^, Michel Salzet^1^****

**^1^**Laboratoire de Spectrométrie de Masse Biologique Fondamentale et Appliquée - EA 4550, Université de Lille 1, Villeneuve d’Ascq, France

^2^ Institute of Neurobiology, Slovak Academy of Sciences, Center of Excellence for Brain Research, University of Veterinary Medicine and Pharmacy, Kosice, Slovakia

*** Equal contribution
** Correspondance**: Michel Salzet: Laboratoire de Spectrométrie de Masse Biologique Fondamentale et Appliquée - EA 4550, Université Lille Nord de France, Bât SN3, 1^er^ étage, Université de Lille 1, F-59655 Villeneuve d’Ascq, France. [Michel.salzet@univ-lille1.fr](mailto:Michel.salzet@univ-lille1.fr)

**Supplementary Material Legends**

**Supplementary Material 1:**Immunofluorescence images of TUJ1 stained (A) and bright field (B) DRG explants exposed to CM LSCI. Note, rich, regularly arranged fine (arrows) and thicker neurite (arrowhead) outgrowth from DRGs. The bright field image shows extension of thin long fibers that are accompanied with three different cells types: spindle-shaped (outlined circle /*) and fibroblast-like cells (outlined circle/ * *) and small round cells (outlined circle /white arrows) (B). (DRG, dorsal root ganglia). Scale bars A = 100 µm; B = 50 µm.

**Supplementary Material 2:**
Summary of the m/z of the different digestion peptides observed, the position in the protein sequence of the digestion peptide for lesion and the databank interrogation (Mascot) score, the sequence coverage and the number of detected digestion fragment observed.

**Supplementary Material 3 & 4:**
Summary of the m/z of the different digestion peptides observed, the position in the protein sequence of the digestion peptide for rostral secretomes and the databank interrogation (Mascot) score, the sequence coverage and the number of detected digestion fragment observed.

**Supplementary Material 5:**Scaffold analysis of MS/MS obtained from control, lesion, rostral and caudal segments.

**Supplementary Material 6:**Immunohistochemistry with anti-Iba1 (red) in thoracic spinal cord sections of sham (A, A´) and rats 3days after injury in rostral segment (B, B´). Increased microglial activation occurred in Gray and White Matter with most prominent manifestation at the lesion site (Dorsal White Matter-DWM). Note, detail of resting microglia with small soma and long thin processes in sham (A´´) and activated microglia with hypertrophied soma after injury (B´´) (A´´, B´´ confocal image) . Scale bars A, B = 300 µm; A´, B´= 10µm

**Supplementary Material 7:**Cross section through rostral spinal cord stained with Iba1 and ED1 antibodies revealing the clear, demarcated lesion site at the dorsal horn white matter (A-D). The core of lesion (asterisk) showed decreased Iba1 IR, while the border zone of penumbra contained accumulated Iba1 positive microglia/macrophages (arrows) (A). Note, the round shaped Iba1 migrating macrophages and activated microglia primarily accumulated within WM (arrows), while Iba1 microglia with hypertrophied somata and thick processes occurred in GM (open arrows) (B). Double immunohistochemistry confirmed that the lesion contained infiltrated ED1 monocytes (green) (C, D). Scale bars A-D= 200 µm.

**Supplementary Material 8:**
Cross section through rostral and caudal spinal cord stained with GFAP antibody from sham (left panel) and SCI (right panel) rats at 3days. Note, the star shaped GFAP astrocytes with long fine processes (arrows) found in sham sections, in contrary to impaired GFAP+ profiles with short processes (arrows) surrounded by debris found in rostral and caudal segments of SCI sections. Scale bar = 100 µm.
