# Supplementary figures and images for: Alterations of protein composition along the rostro-caudal axis after spinal cord injury: proteomic, in vitro and in vivo analyses
Source: Front Cell Neurosci. 2014 Apr 17;8:105. doi: 10.3389/fncel.2014.00105 (PMC4028999; doi:10.3389/fncel.2014.00105)

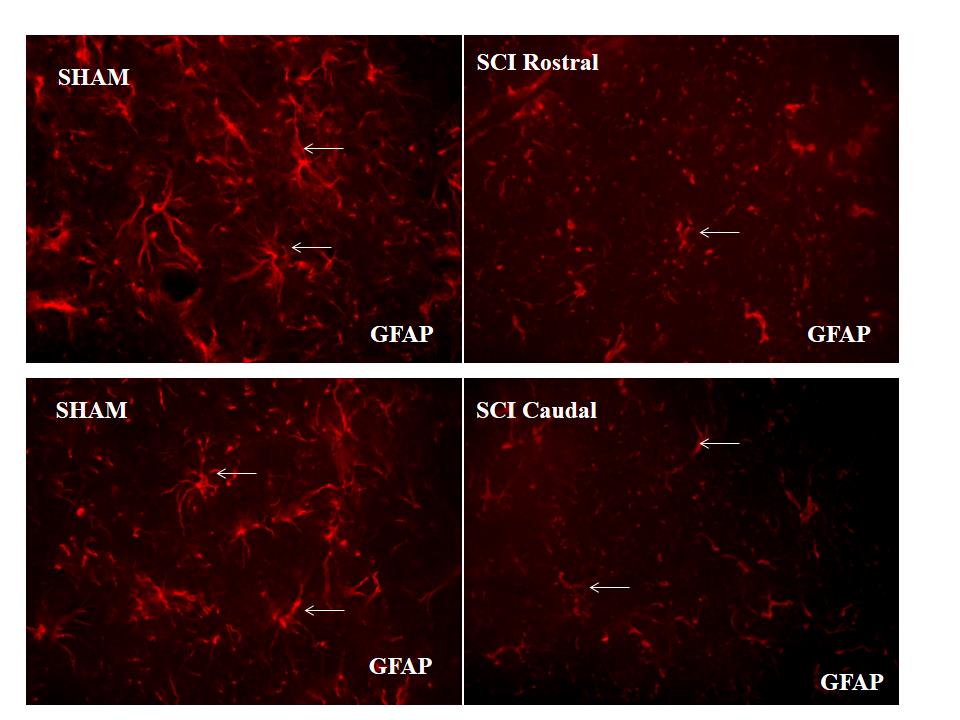

Supplement: Supplementary file 1 [file Presentation1.ZIP › SupplementaryMaterial_8.jpg]

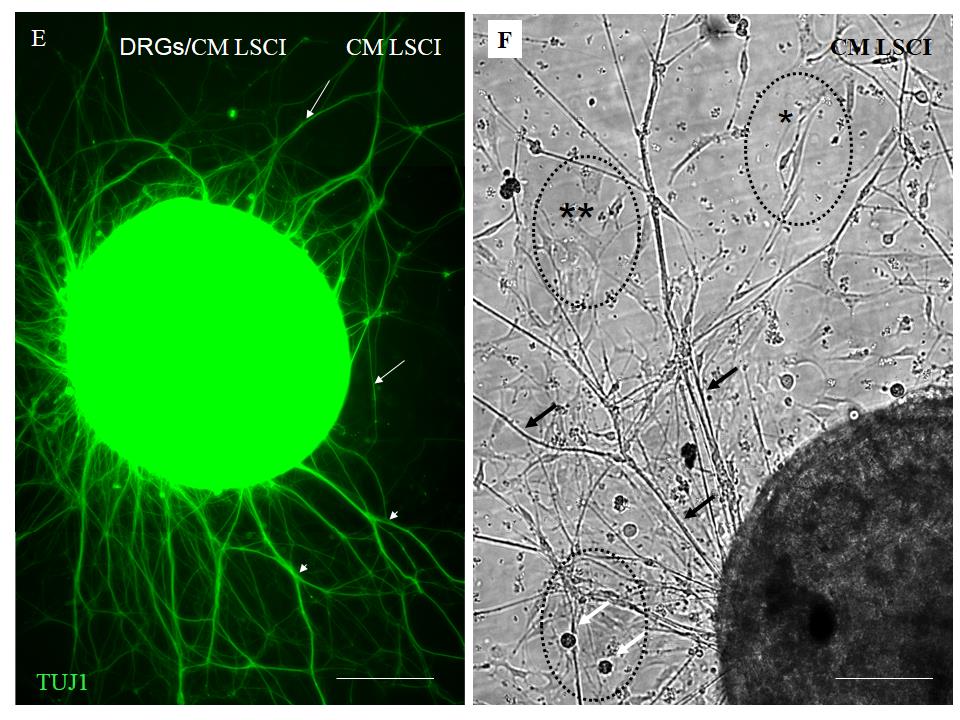

Supplement: Supplementary file 1 [file Presentation1.ZIP › SupplementaryMaterial_1.jpg]

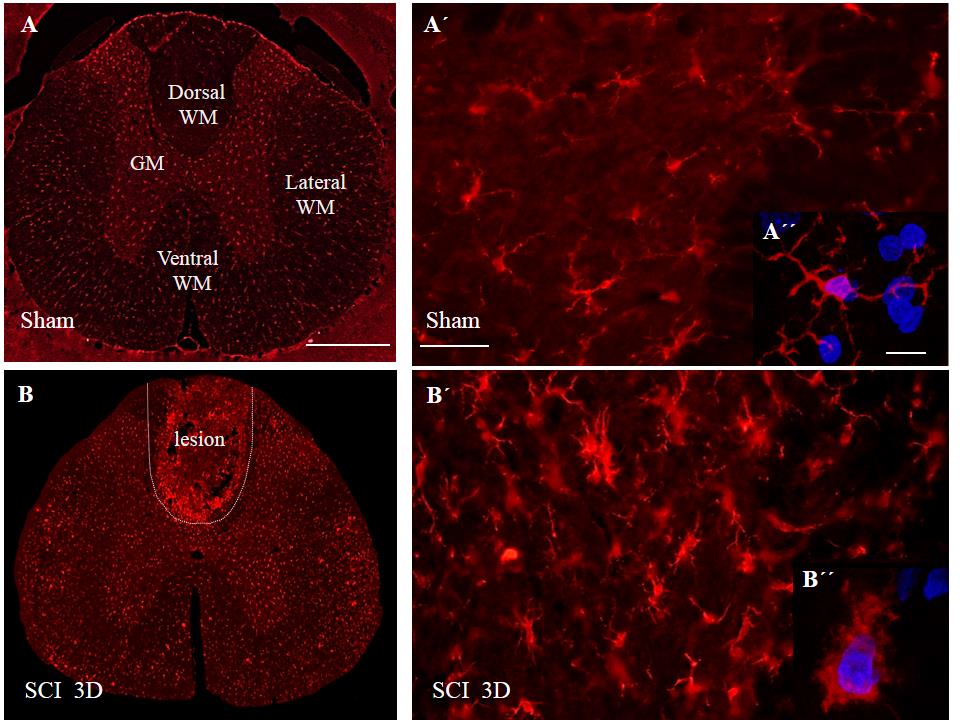

Supplement: Supplementary file 1 [file Presentation1.ZIP › SupplementaryMaterial_6.jpg]

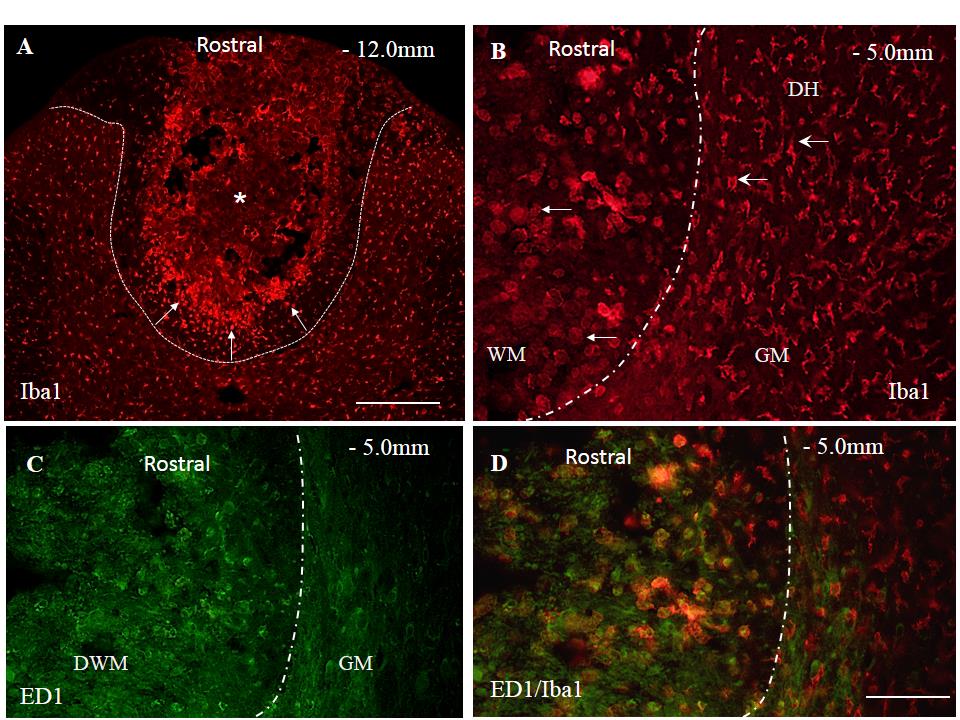

Supplement: Supplementary file 1 [file Presentation1.ZIP › SupplementaryMaterial_7.jpg]
